# Optimizing Rigid Cystoscopy and Biopsy Requests for Red Patches After Flexible Cystoscopy: A Two‑Cycle Quality Improvement Audit

**DOI:** 10.7759/cureus.93060

**Published:** 2025-09-23

**Authors:** Asmita Hossain, Ahmed Tarek Ali Mahmoud Albnhawy, Randeep Dhariwal, Moumn Abdalla, Panagiotis Papikinos

**Affiliations:** 1 Urology, Surrey and Sussex Healthcare National Health Service (NHS) Trust, Redhill, GBR; 2 Urology, University College London Hospitals National Health Service (NHS) Foundation Trust, London, GBR

**Keywords:** bladder biopsy, bladder red patch, flexible cystoscopy, rigid cystoscopy, urothelial bladder cancer

## Abstract

Background

Flat erythematous “red patches” (RPs) identified during flexible cystoscopy are common and often benign, yet they have historically prompted rigid cystoscopy and biopsy under general anesthesia. This practice can lead to unnecessary procedures with low diagnostic yield, added morbidity, and increased healthcare burden.

Objective

To evaluate the local practice regarding rigid cystoscopy and biopsy for RPs and to assess the impact of a targeted departmental intervention.

Methods

This was a single-center, retrospective, two-cycle quality improvement audit at a UK district general hospital. Cycle 1 included all flexible cystoscopies between March and June 2024 and Cycle 2 between November 2024 and February 2025. Data collected included demographics, cystoscopy indication, smoking history, urinary tract infection (UTI) status, biopsy decisions, histology, antibiotic use, and relook outcomes. Following Cycle 1, an intervention was introduced comprising mandatory red-patch image capture, consultant review prior to biopsy listing, and structured teaching on morphology, risk stratification, and benign mimics.

Results

In Cycle 1, 63 RP cases were identified; 37 (58.7%) underwent biopsy, with three malignancies detected (8.1%). Relook cystoscopy was performed in 26 patients, with resolution in 20 (76.9%); of six persistent cases, three underwent biopsy (one malignancy) and three were observed safely. In Cycle 2, 73 cases were identified; 23 (31.5%) underwent biopsy, with one malignancy (4.3%). Relook was performed in 48 patients, with resolution in 32 (66.7%); of 16 persistent cases, eight underwent biopsy (one malignancy) and eight were managed conservatively. Across both cycles, all malignancies occurred in patients >60 years; three of four had a smoking history, and one coincided with proven UTI. Antibiotic prescribing remained frequent, including in patients without culture-proven infection.

Conclusion

In our center, a consultant-led, image-supported, risk-stratified pathway appeared to reduce unnecessary rigid cystoscopy and biopsy requests for RPs, with no observed delays in cancer detection during the audit period. Most lesions resolved spontaneously or after UTI treatment, and several persistent but low-risk patches were managed safely without biopsy. However, with only four malignant cases identified, oncological safety cannot be definitively established, and these findings should be regarded as exploratory. This study is further limited by its single-center, retrospective design and small event rate. Future work should prioritize antibiotic stewardship, standardization of relook intervals, and evaluation of urine cytology in high-risk patients.

## Introduction

Flat erythematous “red patches” (RPs) observed during flexible cystoscopy are a frequent but diagnostically challenging finding. The differential is broad, ranging from carcinoma in situ (CIS) and high-grade urothelial carcinoma to benign conditions such as urinary tract infection, post-instrumentation changes, intravesical therapy, or inflammatory bladder disorders. CIS typically appears as a velvety or granular erythematous lesion, but white-light cystoscopy alone cannot reliably distinguish malignant from benign pathology, leading to variation in biopsy practice. The diagnostic yield of malignancy from RPs is generally low: Swinn et al. reported a 12% malignancy detection rate with no cancers in patients under 60 years [1], Nkwam et al. found a 23.5% malignancy detection rate in surveillance RPs, particularly in intermediate- and high-risk patients [2], and Fernando et al. demonstrated that most RPs in patients investigated for hematuria or lower urinary tract symptoms were benign [3]. These findings support a risk-stratified rather than routine biopsy approach.

Current international guidelines reflect this evidence. The European Association of Urology (EAU) and the American Urological Association (AUA) recommend targeted biopsy of abnormal mucosa primarily in the setting of positive urine cytology or known high-risk non-muscle-invasive bladder cancer [4,5]. Indiscriminate biopsy under general anesthesia increases patient morbidity, theatre workload, and healthcare costs, whereas selective biopsy based on clinical and cystoscopic risk factors improves diagnostic efficiency and safety. Variation in biopsy decision-making across UK centers has also been reported [2], underscoring the need for a standardized, evidence-based pathway. 

RPs at cystoscopy therefore present a diagnostic dilemma; while most are benign, biopsy rates remain high and variable, reflecting clinical uncertainty and the absence of diagnostic methods. To address this gap, we undertook a two-cycle quality improvement audit to evaluate the local practice in the management of RPs, quantify the diagnostic yield of malignancy from biopsies, and assess the impact of a departmental intervention on biopsy requests and cancer detection. The intervention included mandatory photographic documentation, consultant review of images before biopsy listing, and structured teaching to raise awareness of alternative benign causes of red patches. By clarifying practice patterns and outcomes, this study aimed to explore whether a consultant-led, image-supported, risk-stratified pathway could reduce unnecessary biopsies without compromising cancer detection.

## Materials and methods

This was a single-center, retrospective, two-cycle quality improvement audit, acknowledging the possibility of selection bias. It was conducted at a district general hospital in the United Kingdom. The first cycle included all flexible cystoscopies performed between March 1 and June 30, 2024, and the second cycle included those between November 1, 2024 and February 28, 2025. The four-month interval between audit cycles served as the intervention phase, during which departmental changes were introduced in line with established principles of clinical audit and quality improvement methodology [6,7]. 

Patients were eligible if a red patch was documented as the primary cystoscopic finding and either rigid cystoscopy with biopsy was subsequently performed or conservative management was chosen. Exclusion criteria included the presence of visible papillary tumors or solid bladder lesions, incomplete documentation, or cancellation of the procedure due to medical comorbidity. Data were retrieved retrospectively from the electronic patient record system (Cerner accessed through the Citrix Workspace App (Citrix Systems, Inc., Fort Lauderdale, FL, USA)) and the hospital pathology database. Variables collected included patient demographics, smoking history, bladder cancer history, cystoscopy indication, performance status, urine culture and cytology results, biopsy decisions, histology findings, antibiotic use, and outcomes of relook flexible cystoscopy. Data was collated and analyzed using Microsoft Excel (Microsoft 365, Microsoft Corporation, Redmond, WA, USA).

During the intervention period, several measures were introduced to rationalize biopsy requests. A photographic documentation of all red patches at flexible cystoscopy was mandated, and images were automatically incorporated into the patient’s electronic record (Cerner). Consultant review was sought in cases of diagnostic uncertainty, in higher-risk patients, and when biopsies were performed by specialty nurses or registrars. Images were reviewed in real time by the on-call or available consultant before a biopsy decision was made, and the stored photographs also facilitated consultant re-review prior to rigid cystoscopy and biopsy. Structured departmental teaching was delivered to urology staff, focusing on the differential diagnosis of red patches and the endoscopic features that distinguish malignant from benign lesions. Risk stratification was reinforced, incorporating patient age, prior bladder cancer history, long-term catheter use, history of recurrent or active urinary tract infection, and urine cytology status, in line with published guideline recommendations [4,5].

The primary outcomes were the proportion of patients with red patches who underwent rigid cystoscopy and biopsy, and the diagnostic yield of malignancy from those biopsies. Secondary outcomes included the proportion of patients with culture-proven urinary tract infection who received antibiotics, the use and outcomes of relook flexible cystoscopy, and the association between clinical risk factors, source of biopsy request (registrar- or nurse-led), and malignant histology.

This audit was designed to be replicable in other district general hospitals by applying the same inclusion and exclusion criteria, data collection sources, and intervention measures. No sample size calculation or control group was required, as this was a retrospective audit and all eligible patients in each cycle were included in line with standard audit methodology.

## Results

Patient demographics and biopsy requests 

Across the two audit cycles, 136 patients with red patches identified during flexible cystoscopy were included (Cycle 1: 63; Cycle 2: 73). Based on the total number of flexible cystoscopies performed (Cycle 1: 1,197; Cycle 2: 1,168), the proportion of patients with a documented red patch was 5.3% and 6.2%, respectively. 

In Cycle 1, 37 (58.7%) patients were directly scheduled for rigid cystoscopy and biopsy after the initial flexible cystoscopy, compared with 23 (31.5%) in Cycle 2, representing a marked reduction in immediate biopsy requests following the intervention. Demographic details are summarized in Table 1.

**Table 1:** Demographics and clinical history

Characteristic	Cycle 1 (n=63)	Cycle 2 (n=73)
Mean age (years)	65.2	67.1
Male sex, n (%)	48 (76.2)	54 (74)
Smoking history, n (%)	24 (38.1)	30 (41.1)
Known bladder cancer, n (%)	6 (9.5)	11 (15.1)

Histology outcomes and urinary tract infection status 

In Cycle 1, 3/37 biopsies (8.1%) confirmed malignancy, compared with 1/23 (4.3%) in Cycle 2. All malignant cases occurred in patients aged >60 years, most with a smoking history. One was carcinoma in situ (CIS). In Cycle 1, none of the malignant cases had culture-proven urinary tract infection (UTI) at the time of biopsy, while in Cycle 2, one malignancy was diagnosed in a patient with proven UTI. 

Proven UTI was documented in 28 (44.4%) patients in Cycle 1 and 27 (37.0%) in Cycle 2. Despite this, 17 patients (27.0%) in Cycle 1 and seven (9.6%) in Cycle 2 with culture-positive UTI still underwent biopsy; all but one (Cycle 2) yielded benign histology. The comparative outcomes are shown in Table 2.

**Table 2:** Summary of outcomes by audit cycle Percentages for ‘Confirmed malignancy, n (%) of biopsied cases’ reflect biopsy yield (denominator=number biopsied; for example, 3/37 (8.1%)). All other percentages use the total red-patch cohort per cycle as the denominator. UTI: Urinarty tract infection.

Outcome	Cycle 1 (n=63)	Cycle 2 (n=73)	% Change
Biopsy performed, n (%)	37 (58.7%)	23 (31.5%)	↓ 47.0%
Confirmed malignancy, n (%)*	3 (8.1%)	1 (4.3%)	↓ 46.9%
Proven UTI, n (%)	28 (44.4%)	27 (37.0%)	↓ 16.7%
Biopsy+proven UTI, n (%)	17 (27.0%)	7 (9.6%)	↓ 64.4%
Relook performed, n (%)	26 (41.3%)	48 (65.8%)	↑ 59.3%
Resolution after relook, n (%)	20 (76.9%)	32 (66.7%)	↓ 13.3%
Post-relook malignancy, n	1	1	—

Relook flexible cystoscopy and lesion resolution 

Relook flexible cystoscopy was performed in 26 (41.3%) patients in Cycle 1 and 48 (65.8%) in Cycle 2. The interval between initial and relook cystoscopy was not standardized and varied according to clinical judgment and scheduling availability. 

In Cycle 1, red patches resolved in 20 (76.9%) patients, while six patients had persistent lesions. Of these, three underwent biopsy (one malignancy) and three were managed conservatively after consultant review. In Cycle 2, 32 (66.7%) patients showed resolution, while 16 had persistent lesions; eight underwent biopsy (one malignancy) and eight were managed conservatively. Both malignancies diagnosed at relook (one in each cycle) were detected promptly, and no cancers were missed among patients managed without biopsy.

Antibiotics were frequently prescribed. In Cycle 1, 25/26 (96.2%) patients received antibiotics prior to relook, although only 11 (42.3%) had culture-proven UTI. In Cycle 2, 47/48 (97.9%) received antibiotics, with just 18 (37.5%) confirmed infections. One malignancy in each cycle was diagnosed after relook biopsy for a persistent red patch (Cycle 1: four-week delay; Cycle 2: 10-week delay due to recurrent positive cultures); neither delay resulted in adverse outcomes (Table 3).

**Table 3:** Relook cystoscopy outcomes including biopsy vs conservative management of persistent lesions UTI: Urinary tract infection.

Outcome	Cycle 1 (n=26)	Cycle 2 (n=48)
Resolution of red patch, n (%)	20 (76.9%)	32 (66.7%)
Persistent red patch, n	6	16
Biopsied cases (number malignant)	3 (1)	8 (1)
Conservatively managed	3	8
Received antibiotics, n (%)	25 (96.2%)	47 (97.9%)
Proven UTI, n (%)	11 (42.3%)	18 (37.5%)
Post-relook malignancy, n (delay)	1 (4-week delay)	1 (10-week delay)

Characteristics of malignant and suspicious cases 

All malignant or suspicious biopsies occurred in patients aged >60 years; three of the four patients were >65 years. Three patients had a smoking history. Two cases were detected during bladder cancer surveillance, two during hematuria investigation, and one during combined hematuria and lower urinary tract symptoms (LUTS) evaluation. Lesion appearances ranged from discrete raised patches to diffuse erythema. Only one malignant case (Cycle 2) occurred in a patient with proven UTI at the time of biopsy. Two malignancies (one in each cycle) were diagnosed at relook cystoscopy, including one with a 10-week delay due to persistent infection; neither delay led to adverse outcomes. Case details are summarized in Table 4. 

**Table 4:** Summary of confirmed malignant cases UTI: Urinary tract infection; LUTS: lower urinary tract symptoms; RP: red patch.

Case	Cycle	Age (years)	Smoking history	Clinical context	Lesion description	Proven UTI	Diagnosis timing
1	1	>65	Yes	Hematuria+LUTS	Two raised red patches, bladder dome	No	Initial biopsy
2	1	>60	No	Hematuria	Raised red patch, posterior wall	No	Initial biopsy
3	1	>65	Yes	Visible hematuria+LUTS	Erythematous patches, left lateral wall	No	Relook biopsy – persistent RP
4	2	>65	Yes	Surveillance (prior cancer)	Widespread red patches	Yes	Relook biopsy – persistent RP

Urine cytology findings 

In Cycle 1, cytology was not performed in 34 (54.0%) patients. Among those tested, one showed atypical cells, but biopsy revealed only acute inflammation. All other cytology results were negative for malignancy and corresponded to benign histology.

In Cycle 2, cytology was not performed in 48 (65.8%) patients. Two results were inconclusive: one patient had malignant histology, the other benign. One patient had “malignant cells present” on cytology (with known bladder cancer), but the red-patch biopsy was benign. All remaining cytology results were negative and corresponded to benign histology.

**Figure 1:**
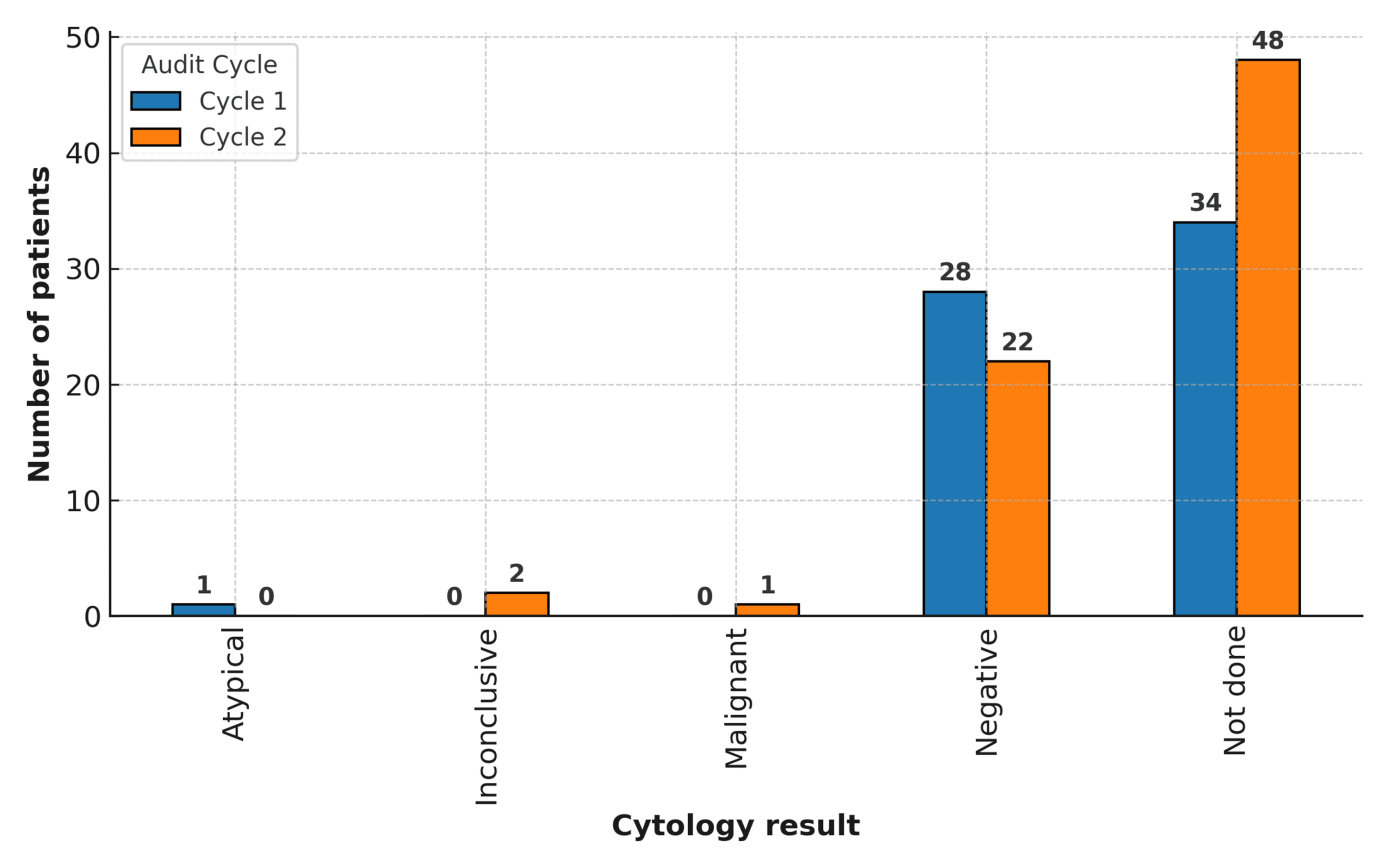
Bar chart comparing the number of patients in each urine cytology result category between Cycle 1 and Cycle 2. Most patients did not have cytology performed. Positive or atypical results were rare and did not consistently correspond to malignant histology.

Overall, cytology in our cohort was often unavailable or non-diagnostic, and positive or atypical findings did not consistently predict malignant histology. No negative cytology result coincided with malignant biopsy, although the small numbers preclude any reliable assessment of rule-out value (Figure 1). Importantly, cytology was not performed routinely, and the data were collected retrospectively. This practice did not appear to follow consistent guidelines and likely reflects a local practice gap, which has now been recognized and will be addressed in future audit cycles.

## Discussion

This audit demonstrated that most RPs detected at flexible cystoscopy were benign, and that a consultant-led, image-supported pathway reduced low-yield biopsy requests. Biopsy rates fell from 37/63 (58.7%) in Cycle 1 to 23/73 (31.5%) in Cycle 2, while malignancy yield decreased from 3/37 (8.1%) to 1/23 (4.3%). Importantly, all malignant cases were detected promptly, suggesting that a selective approach did not compromise safety. These findings are consistent with previous studies: Swinn et al. reported a malignancy yield of 12%, with none in patients younger than 60 years [1]; Nkwam et al. observed a 23.5% malignancy yield in surveillance RPs, particularly among intermediate- and high-risk patients [2]; and Fernando et al. found that most RPs detected during hematuria or LUTS investigations were benign [3]. 

Relook cystoscopy in our series proved to be a safe alternative to immediate biopsy, with two-thirds of lesions resolving spontaneously (Cycle 1: 20/26, 76.9%; Cycle 2: 32/48, 66.7%). No cancers were missed, in line with EAU and AUA recommendations that biopsies should be reserved for persistent or suspicious lesions, especially in the presence of positive cytology or a high-risk history [4,5]. Enhanced imaging modalities, such as narrow-band imaging, have been shown to improve the detection of flat or recurrent lesions, further supporting a selective approach over indiscriminate biopsy [6].

Antibiotics were prescribed frequently, even in the absence of culture-proven infection. In Cycle 1, 25/26 (96.2%) patients undergoing relook cystoscopy received antibiotics, though only 11 (42.3%) had confirmed UTI; in Cycle 2, 47/48 (97.9%) patients received antibiotics, with only 18 (37.5%) confirmed UTI cases. This mirrors wider concerns in urology regarding antimicrobial overuse and highlights the need for adopting a culture-first protocol to strengthen stewardship [7]. 

Urine cytology in our cohort was obtained inconsistently and, in too few patients, to allow meaningful evaluation of its diagnostic utility. In the limited cases available, results were often negative or inconclusive and did not consistently guide biopsy decisions. This limitation is important to acknowledge, and our findings regarding cytology should be considered descriptive only. Existing evidence, however, supports that while cytology has high specificity, it has limited sensitivity for low-grade tumors [8], variable performance for high-grade disease [9], and a risk of false-positives in inflammatory settings [10]. Selective use of cytology in high-risk or persistent RPs may therefore be appropriate, though our data cannot confirm this.

Overall, the intervention nearly halved biopsy requests without missing malignancies. Similar approaches that emphasize structured documentation and cystoscopic image review have been shown to enhance diagnostic accuracy, efficiency, and reproducibility in endoscopic practice [11,12]. Beyond diagnostic yield, reducing unnecessary procedures lessens theater burden and optimizes resource utilization, consistent with broader surgical evidence linking system-level organization and provider volume to improved outcomes [13].

This study has several limitations. It was a single-center, retrospective audit, which introduces selection bias and limits the external validity of the findings. The number of malignant cases detected was very small (n=4 across both cycles), and with such a low event rate, formal statistical testing was not performed; our results are therefore descriptive and should be regarded as exploratory and hypothesis-generating rather than definitive. The oncological safety of deferring biopsies cannot be confirmed, and our conclusions are framed in terms of feasibility and potential safety. The interval between initial and relook flexible cystoscopy was not standardized, reflecting real-world practice variation. This variability may have influenced the timing of lesion resolution and detection of malignancy and represents an additional limitation of the study. Cytology was not obtained routinely in our cohort, particularly among patients coming to the hospital for the surveillance of known bladder cancer, and inconclusive results were not always repeated. These patterns reflect local practice rather than guideline-based application. As cytology data were collected retrospectively, its limited and inconsistent use represents a significant limitation, and our findings regarding cytology must therefore be considered exploratory. We plan to present these results within our department to inform a more structured approach to cytology use in future. Lesion photography was inconsistently applied in the first cycle, though standardized in the second. True validation would require larger, prospective multicenter studies. Despite these methodological constraints, quality audit provides valuable quality improvement insights, demonstrating how structured departmental interventions can rationalize biopsy requests and align local practice more closely with international guidelines.

Future work should prioritize embedding mandatory photographic documentation into all cystoscopy procedures, requiring consultant approval before biopsy listing, and adopting a culture-first antibiotic protocol. Development of a formal risk-stratification tool incorporating age, smoking history, prior bladder cancer, lesion morphology, and cytology may further refine biopsy decisions. Prospective multicenter studies with standardized lesion reporting, consistent follow-up, and the use of enhanced visualization techniques such as narrow-band imaging or photodynamic diagnosis would provide more robust evidence to support selective biopsy strategies. 

In summary, this quality audit supports a selective, risk-stratified approach to the biopsy of red patches at flexible cystoscopy. Such a pathway reduced unnecessary procedures, maintained cancer detection, aligned with international guidelines, and identified opportunities to improve antimicrobial stewardship, diagnostic standardization, and healthcare efficiency.

## Conclusions

This two-cycle audit demonstrated that most red bladder patches identified at flexible cystoscopy were benign. In our center, a consultant-led, image-supported, risk-stratified pathway appeared to reduce unnecessary rigid cystoscopy and biopsy requests without any observed delay in the detection of clinically significant cancer. Most lesions resolved spontaneously or after treatment of urinary tract infection, and several persistent but low-risk patches were safely managed without biopsy following consultant review. However, with only four malignant cases identified, statistical analysis was not feasible, and oncological safety cannot be definitively established. These findings should therefore be interpreted as exploratory and hypothesis-generating, reflecting feasibility and potential safety, rather than as definitive proof. Future work should prioritize the standardization of relook cystoscopy intervals, incorporation of urine cytology in high-risk patients, improved antimicrobial stewardship, and prospective multicenter evaluation to validate selective biopsy strategies.
